# 胸膜间皮瘤相关小肠受累致回肠穿孔1例并文献复习

**DOI:** 10.3779/j.issn.1009-3419.2026.106.16

**Published:** 2026-06-20

**Authors:** Yunjia WANG, Fangqian SHEN, Qisha LI, Xinjuan LI, Hua YANG, Puyu LIU, Xuefeng YANG, Nianjie ZHANG, Jianguo ZHOU, Yuju BAI

**Affiliations:** ^1^563000 遵义，遵义医科大学第二附属医院胸部肿瘤科（王韵佳，申芳倩，李启莎，周建国，柏玉举）; ^1^Department of Thoracic Oncology; ^2^影像科（李信娟）; ^2^Department of Radiology; ^3^病理科（杨华，刘璞玉）; ^3^Department of Pathology; ^4^胃肠外科（杨雪峰，张念杰）; ^4^Department of Gastrointestinal Surgery, The Second Affiliated Hospital of Zunyi Medical University, Zunyi 563000, China

**Keywords:** 胸膜间皮瘤, 小肠转移瘤, 穿孔, 贝伐珠单抗, Pleural mesothelioma, Small intestinal metastasis, Perforation, Bevacizumab

## Abstract

胸膜间皮瘤（pleural mesothelioma, PM）通常以胸腔内局部浸润为主，小肠受累罕见。本文报道1例37岁男性初诊IV期弥漫性PM患者，除长期吸烟史外无明确石棉及其他特殊职业或环境暴露史。患者经培美曲塞、卡铂联合贝伐珠单抗一线治疗后疾病稳定，维持治疗期间突发急腹症，急诊手术证实回肠末端穿孔。术后病理提示PM相关回肠受累/转移性受累，Ki-67增殖指数较原发灶升高。因仅行穿孔修补而非肠段切除，现有材料不能判断肠壁全层受累及浸润方向；结合影像、术中所见及病理结果，暂不支持原发性腹膜间皮瘤或膈肌病灶直接连续侵犯回肠，但不能完全排除微小腹膜播散继发肠壁受累。本文将结合文献复习总结PM相关小肠受累的诊断难点、处理策略及临床启示。

## Abstract (en)

Pleural mesothelioma (PM) usually manifests as local intrathoracic invasion, and small intestinal involvement is rare. A 37-year-old man with initially diagnosed stage IV diffuse PM and no definite history of asbestos exposure or other specific occupational or environmental exposure except for long-term smoking was reported. After first-line treatment with Pemetrexed, Carboplatin and Bevacizumab, the disease remained stable. However, acute abdomen occurred during maintenance therapy, and emergency surgery confirmed terminal ileal perforation. Postoperative pathology suggested PM-related ileal involvement/metastatic involvement, with a higher Ki-67 proliferation index than that of the primary lesion. Because only perforation repair rather than segmental bowel resection was performed, the available pathological material could not determine whether full-thickness bowel-wall involvement was present or the direction of tumor invasion. Imaging findings, intraoperative findings and pathological results did not support primary peritoneal mesothelioma or direct contiguous invasion of the ileum by the diaphragmatic lesion; however, microscopic peritoneal dissemination with secondary bowel-wall involvement could not be completely excluded. The aim of this report, together with literature review, is to summarize the diagnostic challenges, management strategies and clinical implications of PM-related small intestinal involvement.

胸膜间皮瘤（pleural mesothelioma, PM）是一种少见的侵袭性肿瘤，起源于胸膜间皮细胞，多数患者确诊时已为晚期，预后较差^[1,2]^。PM主要表现为胸腔内局部浸润，随着疾病进展可发生胸腔外受累，其中消化道受累较少见，小肠受累或转移尤为罕见^[3,4]^。小肠继发性肿瘤可引起消化道出血、肠梗阻或肠穿孔等外科急症，部分患者需急诊手术处理^[5]^。本文报道1例PM相关回肠受累致穿孔病例，并结合文献讨论其识别与处理要点。

## 1 病例资料

患者男，37岁，既往史无特殊，有长期吸烟史。进一步追问流行病学及职业暴露史：除长期吸烟史外，未诉明确石棉接触史，亦无建筑/装修、矿山粉尘、造船、锅炉、石棉水泥制品、隔热/保温材料等相关职业或环境暴露史，无明确胸部放射性暴露史。2024年4月因“胸痛不适”就诊，胸部计算机断层扫描（computed tomography, CT）显示右上纵隔旁肿物，磁共振成像（magnetic resonance imaging, MRI）显示肿物侵犯胸椎、肋骨、纵隔、膈肌和肺（图1A）。穿刺活检组织学可见上皮样肿瘤细胞，结合免疫组化及影像学表现，诊断为PM，上皮样型。免疫组化结果：细胞角蛋白（cytokeratin, CK）（+++）、钙视网膜蛋白（calretinin, CR）（+/-）、细胞角蛋白5/6（cytokeratin 5/6, CK5/6）（+/-）、D2-40（podoplanin）（-）、Wilms肿瘤1蛋白（Wilms tumor 1, WT-1）（-）、MOC-31（monoclonal antibody）（-）、细胞角蛋白7（cytokeratin 7, CK7）（-）、甲状腺转录因子-1（thyroid transcription factor-1, TTF-1）（-）、天冬氨酸肽酶A（novel aspartic proteinase A, Napsin A）（-）、P40（-）、Ki-67（10%+）（图2）。因标本为穿刺组织，未能完整评价结构模式、核分级、坏死及少量肉瘤样成分。结合多部位胸腔内外浸润及转移表现，本例符合弥漫性PM，临床分期为cT4N1M1，IV期。

**图 1:**
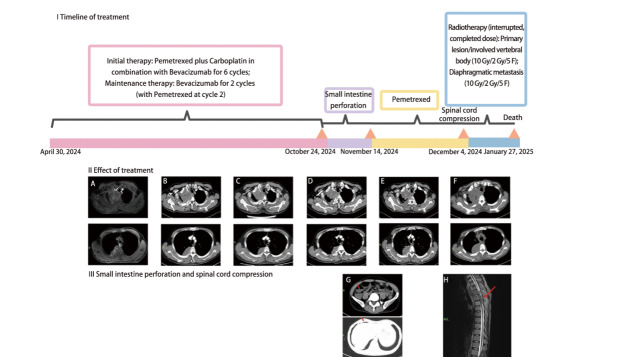
患者诊疗时间线及治疗过程中影像学动态变化。I：诊疗时间线。II：原发胸膜病灶的基线及治疗后影像学表现：A：基线时右上纵隔旁胸膜病灶；B-D：培美曲塞+卡铂+贝伐珠单抗治疗2、4、6个周期后；E：贝伐珠单抗维持治疗1个周期后；F：维持治疗3个周期后（第1个周期：贝伐珠单抗；第2个周期：培美曲塞+贝伐珠单抗；第3个周期：培美曲塞）。III：治疗过程中并发症相关影像学表现：G：腹部CT提示气腹及小肠穿孔相关表现；H：胸椎受侵相关MRI表现。

**图 2:**
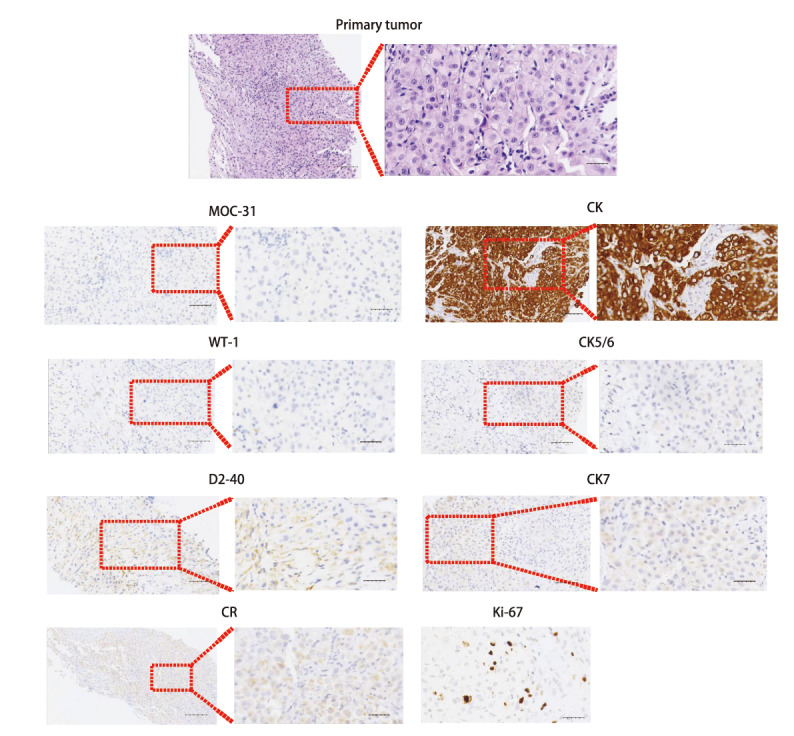
原发胸膜病灶的组织病理学和免疫组织化学特征。HE染色显示肿瘤组织形态，放大倍数分别为×100和×400；IHC染色放大倍数分别为×200和×400。

鉴于患者为IV期PM，无手术根治指征，于2024年4月30日予培美曲塞、卡铂联合贝伐珠单抗一线治疗：培美曲塞800 mg，第1天；卡铂500 mg，第1天；贝伐珠单抗800 mg，第1天；每21天为1个周期。患者共接受6个周期治疗，按实体瘤疗效评价标准1.1版（Response Evaluation Criteria in Solid Tumors version 1.1, RECIST 1.1）评价为疾病稳定（stable disease, SD）（图1B-1D），随后进入维持治疗阶段，单用贝伐珠单抗800 mg治疗1个周期，使用培美曲塞800 mg联合贝伐珠单抗800 mg维持治疗1个周期，以上两周期均每21天为1个周期（图1E-1F）。

维持治疗期间，患者于2024年10月24日突发持续性剧烈腹痛，急诊腹部CT示气腹、部分肠管扩张、积气及积液（图1G），白细胞和中性粒细胞升高，考虑急性消化道穿孔。急诊行腹腔镜探查术，见回肠末端距回盲部约100 cm处穿孔。腹腔内未见明显腹水、腹膜或网膜结节及弥漫性种植灶，术中记录未提示膈下病灶与肠管直接粘连或连续侵犯；其余小肠及结肠未见新生物，遂行回肠穿孔修补术。

术后病理示回肠穿孔周围组织可见上皮样肿瘤细胞浸润。因手术方式为穿孔修补而非肠段切除，现有切片不能完整显示黏膜、黏膜下层、肌层及浆膜层连续结构，故不能判断是否为全层浸润及浸润方向。结合胸膜原发灶病理、影像学表现、回肠病灶部位及术中所见，考虑PM相关小肠受累/转移性受累（上皮样型），暂不支持原发性腹膜间皮瘤直接累及小肠或膈肌病灶直接连续侵犯回肠，但不能完全排除微小腹膜种植继发肠壁受累。免疫组化结果：CR（+）、WT-1（+）、CK5/6（+）、间皮细胞标志物（mesothelial cell marker, MC）（+/-）、上皮膜抗原（epithelial membrane antigen, EMA）（+）、波形蛋白（vimentin）（+）、TTF-1（-）、D2-40（-/+）、平滑肌肌动蛋白（smooth muscle actin, SMA）（-）、结蛋白（desmin）（-）、Napsin-A（-）、Ki-67（60%+）（图3）。

**图 3:**
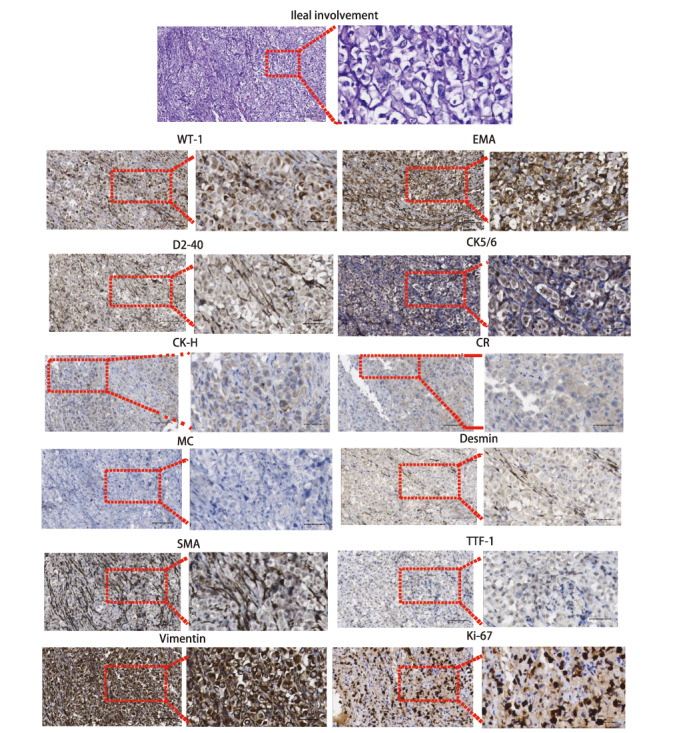
回肠受累病灶的组织病理学和免疫组织化学特征。HE染色显示肿瘤组织形态，放大倍数分别为×100和×400；IHC染色放大倍数分别为×200和×400。

术后患者恢复尚可，但随后肿瘤进展。因经济原因，患者拒绝纳武利尤单抗或伊匹木单抗等免疫治疗，于2024年11月14日续予培美曲塞单药挽救性化疗。2024年12月4日患者复查影像提示胸椎受侵病灶进展（图1H）；患者出现双下肢感觉异常等神经系统症状，临床考虑存在脊髓压迫相关表现，具备手术评估指征，但患者拒绝手术治疗，遂予局部放射治疗。计划剂量为原发胸廓上缘病灶及受侵椎体40 Gy/20 F，膈面转移瘤50 Gy/25 F。放疗期间症状加重并出现双下肢感觉消失（实际照射剂量均为10 Gy/2 Gy/5 F），患者放弃放疗并要求出院。2025年1月27日患者因严重肺部感染死亡。

本研究为病例报道，已获得遵义医科大学第二附属医院伦理委员会豁免审查。患者相关临床资料、影像学资料及病理图片均已进行隐私匿名化处理。患者已死亡，病例资料及相关图片发表已取得其近亲属/法定代理人的书面知情同意。

## 2 讨论

石棉接触是PM最明确的危险因素之一，可见于建筑/装修、矿山、造船、锅炉、石棉水泥制品及隔热/保温材料等职业或环境场景。本例除长期吸烟史外，无明确特殊职业或环境暴露史，亦无胸部放射线暴露史。吸烟并非PM的典型独立危险因素，提示本例病因学线索尚不明确。

PM传统上以胸腔内局部浸润为主，但尸检研究显示晚期病例胸腔外播散并不少见。小肠上皮更新快、DNA修复机制较强，且腔内环境不利于肿瘤种植，因此PM相关小肠受累或转移临床少见，多以个案报道出现^[6-8]^。

既往9例PM相关小肠受累或转移病例报道^[7-15]^和本例共10例患者中，年龄范围37-73岁，中位年龄约59.5岁；既往9例中，其中6例均为女性，3例为男性，本例也为男性。临床表现以急腹症或消化道症状为主，包括肠套叠3例、肠穿孔4例（含本例）、消化道出血或贫血3例。受累部位以空肠最常见，其次为十二指肠和回肠；7例经外科手术确诊，3例经内镜相关检查发现，提示此类病变常在出现严重并发症后才被识别。

PM患者随访通常聚焦胸腔内病灶，小肠受累早期症状缺乏特异性，腹痛、呕吐、贫血或消化道出血易被误认为治疗相关不良反应。对新发腹部症状、消化道出血、贫血、感染指标升高或腹膜刺激征者，应及时行腹部增强CT，必要时结合正电子发射计算机断层显像（positron emission tomography/CT, PET/CT）、胶囊内镜或双气囊小肠镜；若出现气腹、梗阻、套叠或活动性出血，应优先外科/介入处理并取得病理标本。

本例穿孔前无明确持续腹部症状或消化道出血记录，提示回肠受累灶可能短期进展并发生坏死或穿孔。因未行完整肠段切除，不能确认肠壁受累层次和浸润方向，故本文采用“PM相关小肠受累/转移性受累”的谨慎表述。

文献^[9,10]^显示，继发性小肠受累患者总体预后较差，中位生存期较短；表1^[7,8,15-21]^可获得数据的PM病例亦提示，一旦出现穿孔、梗阻、套叠或出血，预后不佳。治疗上应先处理急症并取得病理依据，随后根据全身疾病负荷、体能状态、既往治疗、病灶范围和患者意愿选择姑息手术、系统治疗、放疗或最佳支持治疗。

**表 1:** 恶性胸膜间皮瘤小肠转移的既往病例报告

Author	Year	Age (yrs)	Sex	Treatment	Small intestinal involvement/metastasis					Time from small intestinal involvement/metastasis to death (yr)	Overall survival (mon)
					Clinical manifestation	Symptoms	Site	Diagnostic method	Interval* (mon)		
Prior et al.^[15]^	1993	53	Female	None	Intussusception	Abdominal pain	Not reported	Surgery	7	1	8
Kakugawa et al.^[8]^	2007	62	Female	Surgery	Gastrointestinal bleeding	Anemia	Not reported	Endoscopy	9	Not reported	Not reported
Chen et al.^[16]^	2008	73	Male	None	Gastrointestinal bleeding	Anemia	Duodenum	Endoscopy	0	1	1
Liu et al.^[17]^	2010	57	Female	Surgery and chemotherapy	Intussusception	Abdominal pain and vomiting	Jejunum	Surgery	10	1	11
Gocho et al.^[18]^	2010	52	Female	Chemotherapy	Intestinal perforation	Abdominal pain	Jejunum	Surgery	0.23	12	12
Martínez et al.^[19]^	2010	57	Female	Not reported	Intestinal ulcer	Abdominal pain and anemia	Duodenum	Endoscopy	0	Not reported	Not reported
Navarro et al.^[20]^	2015	67	Female	Surgery and chemotherapy	Intestinal perforation	Abdominal pain	Jejunum	Surgery	13	Not reported	Not reported
Alkhayal et al.^[21]^	2016	65	Male	Not reported	Intestinal perforation	Abdominal pain and vomiting	Jejunum	Surgery	0	Not reported	Not reported
Hamaoka et al.^[7]^	2021	72	Male	Chemotherapy and immunotherapy	Intussusception	Abdominal pain	Jejunum	Surgery	18	Not reported	Not reported
This case	2024	37	Male	Chemotherapy and targeted therapy	Intestinal perforation	Abdominal pain	Ileum	Surgery	6	3	9

*Interval from the initial diagnosis of pleural mesothelioma to the detection of small intestinal involvement/metastasis.

本例回肠穿孔可能与肿瘤相关肠壁受累、局部缺血坏死及贝伐珠单抗相关穿孔风险共同有关。胃肠道穿孔为贝伐珠单抗少见但严重的不良反应，文献^[11-13]^报道发生率为0.9%-4%，多数发生于初始给药后6个月内。因此，对接受抗血管生成治疗的PM患者，如出现腹痛、感染指标升高或不明原因消化道症状，应及时完善腹部影像并重新评估继续用药的风险-获益。

PM相关腹腔内受累机制尚不明确，既可能与肿瘤经膈肌直接延伸进入腹膜腔有关，也可能与腹膜播散继发肠壁受累或少见的胸腔外转移有关^[14]^。本例术中未见弥漫性腹膜病变，回肠穿孔部位与膈肌病灶解剖上不连续，暂不支持膈肌病灶直接连续侵犯回肠；但受取材限制，仍不能排除微小腹膜种植后由浆膜面累及肠壁。

本例为IV期弥漫性PM（上皮样型）患者，在贝伐珠单抗联合化疗后发生回肠穿孔，病理提示PM相关小肠受累/转移性受累。现有证据暂不支持原发性腹膜间皮瘤或膈肌病灶直接连续侵犯回肠，但不能完全排除微小腹膜播散后继发肠壁受累。该病例提示，PM患者出现腹痛、贫血、消化道出血或感染指标升高时，应警惕小肠受累及穿孔风险，尤其在接受抗血管生成药物治疗期间。早期识别、及时处理急症、获取病理证据并个体化选择后续治疗，是改善此类罕见并发症管理的关键。
